# Evaluation of sterility testing procedures for laboratory animal rodent diets

**DOI:** 10.1016/j.vas.2026.100570

**Published:** 2026-01-14

**Authors:** Jonathan W. Weeks, Jacqueline Locklear, Tanya E. Whiteside, David M. Kurtz

**Affiliations:** Quality Assurance Laboratory, Comparative Medicine Branch, National Institute of Environmental Health Sciences, Research Triangle Park, NC, USA

**Keywords:** *Abbreviations and Acronyms:* BAP, blood agar plate, CDC, Centers for Disease Control, FDA, United States Food and Drug Administration, MNV,Mouse Norovirus, MPV, Mouse Parvovirus, NIEHS, National Institute of Environmental Health Sciences, PBW, phosphate buffered water, QAL, Quality Assurance Laboratory, TG, Tryptone glucose, USDA, United States Department of Agriculture

## Abstract

The United States Food and Drug Administration (FDA) and the United States Department of Agriculture (USDA) recommend grinding or stomaching food for human consumption prior to Salmonella testing. The Quality Assurance Laboratory (QAL) at the National Institute of Environmental Health Sciences (NIEHS) routinely tests all incoming, unsterilized rodent feed for *Salmonella spp*. and total microbial load prior to use. Currently, the QAL cultures intact feed pellets in sterile Thioglycolate enrichment broth that sits stationary in the incubator for seven days. Under these conditions, a natural ingredient pelleted feed does not break down thoroughly possibly preventing detection of viable microbes at the pellet center. The purpose of this study was to determine if grinding pelleted rodent feed improves the sensitivity of microbial detection. Our study compared bacterial growth from an unautoclaved and autoclaved, natural ingredient rodent diet (NIH-31) processed as intact pellets or ground feed. Our results indicate that grinding unautoclaved feed for total aerobic bacteria testing does increase the sensitivity of microbial detection. However, when the feed is appropriately sterilized, grinding the feed provides no added benefit.

## Highlights

• Salmonella screening and sterility testing of animal feed is carried out in Quality Assurance Labs.
• Alternative method for evaluating microbial sensitivity in animal feed testing.
• Agitation adds no advantage when testing properly sterilized animal feed.
• Grinding unautoclaved feed increases the sensitivity for aerobic bacteria.
• Grinding properly autoclaved feed is unnecessary when performing sterility testing.

## Introduction

Microbial contamination of food is a common and ongoing problem for humans and animals. Pathogenic contaminants such as Salmonella species can cause severe disease in animals, lead to significant economic impacts in food animals, and make their way into the human food supply (Centers for Disease Control and Prevention CDC, 2020; Crump et al., 2002). The United States Food and Drug Administration (FDA) conducts surveys of animal feed and feed ingredients. FDA conducted a survey in 1993 from 78 feed mills and detected *Salmonella enterica* in 56 % of the 101 animal protein based feed samples tested and 25 % of 89 finished feed samples collected from feed mills in 1994 (Crump et al., 2002). In the same 1993 FDA survey, *Salmonella enterica* was detected in 36 % of the 50 vegetable protein based feed samples collected from 46 feed mills (Crump et al., 2002). The findings show animal feed is frequently contaminated with pathogenic bacteria. From an animal health surveillance perspective, Salmonella is a familiar bacterium of concern and has been reported as a contaminate in United States animal feed as early as 1948 (Crump et al., 2002). Several outbreaks have been reported from animal feed resulting in human food borne illness caused by *Salmonella enterica* (Crump et al., 2002). The source of food borne illness has been known to be by the transmission of contaminated feed to the animals including cattle, pigs, chickens, turkeys, and mice. Consequently, there was an outbreak of Salmonella infections in people linked to canine treats in which the FDA and Centers for Disease Control (CDC) investigated and advised consumers to avoid and retailers to stop sales of such treats (Food & Drug Administration, 2024). In similar cases, the FDA had to require pet food recalls due to Salmonella and Listeria contamination in popular pet food brands (Blue Ridge Beef, 2025; Mars Petcare US, inc February 13, 2025). Non-sterile, laboratory animal feeds produced by reputable feed manufactures should not be assumed to be free of microbial pathogens, especially natural-ingredient, grain-based feeds. The use of non-sterile, rodent feed has been implicated in an outbreak of mouse parvovirus (MPV), a rodent pathogen of concern (Watson, 2013). MPV and Mouse norovirus (MNV) have been shown to survive standard feed pelleting processes (Adams et al., 2019). We have isolated *Clostridium perfringens*, a human and animal pathogen, from multiple, non-sterile, natural-ingredient, rodent diets (Johnston et al., 2022). Colleagues at another NIH institution, although data is not published, reported Salmonella contamination of a natural-ingredient rodent diet resulting in mortality.

The safety of animal feed is regulated by several agencies including the FDA, US Department of Transportation under the Sanitary Food Transportation Act of 1990, and United States Department of Agriculture (USDA) Animal and Plant Health Inspection Service (USDA, Food Safety and Inspection Service, 2024). Such safety measures are enforced and regulated through the Food Safety Modernization Act (FDA, 2011), Federal Food, Drug, and Cosmetic Act (United States Congress, 1938), and the Current Good Manufacturing Practices (FDA CGMP, 2025). For this reason, commercial animal feed poses risks of contamination with pathogens, mycotoxins, and chemical residues. Thus, preventive practices are conducted as set by federal guidelines and standard microbiological procedures to monitor and limit contamination. Having a better understanding and control of Salmonella involves using microbiological analytical methods in which both the FDA and the USDA outline test methods for the testing of human food for Salmonella contamination (Analytical Manual (BAM), 1998; Sargeant et al., 2021; Watson, 2013). We routinely test all rodent diets for Salmonella contamination.

Sterilization of human food and/or animal feed is one method used to eliminate microbial contamination. Due to animal feed being manufactured differently than human food, it has been suggested that bacterial contamination can be significantly reduced or eliminated with steam (65 to 80 °C) during the pelleting process (Adams et al., 2019; Furuta et al., 1980). For human food, sterilization by exposure to gamma irradiation is common (Asmarani et al., 2024; Blue Ride Beef, 2025; FDA, 2011; Hashim et al., 2024; Indiarto et al., 2023; Kim et al., 2024; Marcu et al., 2013; Mrityunjoy et al., 2019). For example, one study looked at gamma irradiation in ground beef to reduce a Shiga toxin-producing *Escherichia coli*.^5^(Cap et al., 2021) Another looked at sterilization in poultry and found that gamma irradiation can eliminate food borne pathogens such as Salmonella, aerobic bacteria, coliform and yeast (Asmarani et al., 2024). Irradiation of laboratory animal feeds at common exposures of 25–50 kGy does not guarantee sterility and can result in the oxidation of unsaturated fatty acids and the destruction of certain vitamins (Vit A and Vit K) (Sargeant et al., 2021). Pasteurization, a commonly used practice for human food, uses lower temperatures (approximately 107 °C) (Caulfield et al., 2008) to destroy non-spore forming bacteria, including Salmonella but is not a common practice with laboratory animal feeds (Adams et al., 2019; Kurtz et al., 2018). Just like there are contamination concerns with commercial animal feed, there are concerns with laboratory animal feed where the foundational goal is to maintain the health and welfare of the animals. Any contamination can introduce extrinsic variables that can compromise the integrity and reproductivity of research data, animal welfare, and researcher safety. Thus, sterilization of animal feed is crucial in maintaining animal welfare as it lessens the likelihood of pathogen introduction which can sicken lab animals or worst, lead to the loss of entire animal colonies.

In the laboratory animal field, animal diets are routinely sterilized using steam sterilization via an autoclave or irradiation (Kurtz et al., 2020). The high temperatures, 230°F to 270°F used in steam autoclaves destroy heat-liable vitamins (Kurtz et al., 2018; Twaddle et al., 2004); therefore, feed manufactures routinely add additional heat-liable vitamins (the exact amount of extra vitamins depends on the specific sterilization method, autoclaving or irradiation and the potential loss of nutrients during that process) including Vit A and Vit B1, B6, B12, E, thiamine, pantothenic acid, and pyridoxine to assure proper concentrations post-sterilization (Indiarto et al., 2023; Marcu et al., 2013; Reinhard, 1996). The National Institute of Environmental Health Sciences (NIEHS) routinely autoclaves their protein based NIH-31 diet. All incoming feeds are tested for microbial pathogens prior to use. All non-sterile diets are also tested for total microbial burden. All feed sterilization is confirmed by culture of intact feed pellets in Thioglycolate enrichment broth follow by plating onto Trypticase Soy agar with 5 % sheep blood. The FDA and USDA protocols for human food recommend grinding the food prior to microbial testing, and most food for human consumption is not sterile. At NIEHS, our current protocol for testing feed sterility post autoclave omits grinding animal feed.

The primary objective of this study was to compare the sensitivity of microbial detection between intact, pelleted and ground rodent feed. Since the intact feed pellets do not completely break down using our current method of sterility testing, we hypothesized that grinding would improve the detection of bacteria that may have survived the autoclaving process. We also included a group of intact, pelleted feed that was gently swirled in the broth cultures throughout incubation to allow for better pellet breakdown compared to stationary broth cultures of intact pelleted feed.

## Materials & methods

### Study design. animal feed

This study used the open formula, natural ingredient NIH-31 (Hutton et al., 1985), autoclavable rodent formulation (Zeigler Brothers, Inc., Gardners, PA) divided into the following experimental groups.

1. Unautoclaved ground feed (UG)
2. Unautoclaved pelletized feed (UP)
3. Autoclaved ground feed (AG)
4. Autoclaved pelletized feed (AP)

### Autoclave parameters

The pellets are oval shaped, 5/8 inches wide, 3/8 inches thick, and ½ to one inch long. The NIH-31 pellets were autoclaved in 25 lb. paper bags in a Model RSP AMSCO bulk autoclave (Steris Life Sciences, Mentor, OH). The autoclave cycle consisted of three pre-vacuum purges, a 20-minute exposure period at 250° F (121° C), 15 to 18 lbs. of pressure, and one 10-minute drying period at 250°F (Kurtz et al., 2018).

### *Experimental procedures.* microbial testing with total aerobic plate counts

The total aerobic bacterial plate counts were determined by using standard microbiological procedures (Carter, 1973).^6^ This method allows for a direct numerical comparison of bacteria present between the two feed matrices (ground vs pelleted). Using aseptic technique, 0.025 kg of UP and AP feeds were placed into a sterilized Waring commercial blender 7011HS (model HGB2WTS3 Torrington, CT) and ground on the counter. The blender was then transferred underneath the Class II Type A2 biological safety cabinet to prevent cross contamination. Twenty-five thousandths of a kilogram of each feed (UG, UP, AG, and AP) were placed into 100 mL of sterile phosphate buffered water (PBW: 1st dilution bottle) and each feed bottle sample was gently swirled to mix. After 10 min of settling time in the PBW, 1.0 mL of dilution bottle 1 mixture was transferred to a 2nd, 100 mL sterilized PBW dilution bottle and gently swirled to mix. Serial dilutions were aliquoted into the center of sterile, 100 mm petri dishes by pipetting 1 mL, 0.1 mL from PWB 1 and 1 mL, 0.1 mL from PWB 2 (Carter, 1973; Salfinger, 2015; Sanders, 2012). Sterile tryptone glucose (TG) agar was prepared by boiling to liquify and then placed in 55 °C water. The warm, liquid TG agar was poured over the samples and mixed using a circular figure eight pattern. After media solidification, plates were inverted and incubated aerobically at 37 °C for 48 h before counts were determined. All plate counts were performed in triplicate for each group.

### Sterility testing of feed

**Fig. 1:**
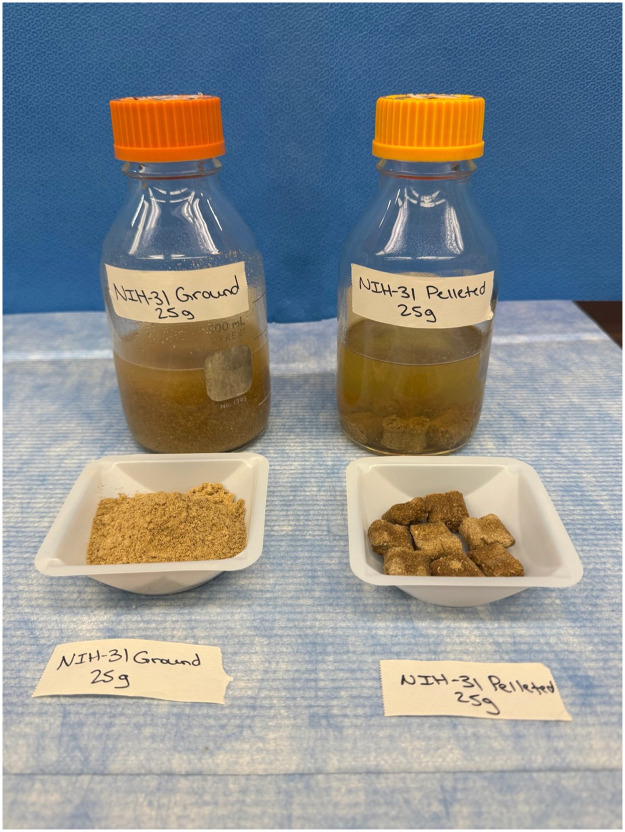
NIH-31 diet in Thioglycolate broth: ground versus pelleted.

**Fig. 2:**
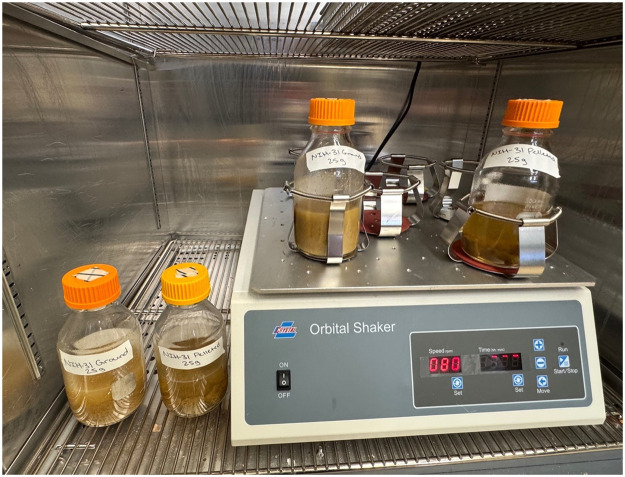
Sterility testing setup for pelleted and ground NIH-31 diet in Thioglycolate broth incubated at 37 °C illustrating stationary bottles (left) and bottles on the orbital shaker set at 80 rpm (right).

Using aseptic technique, 0.025 kg of each diet (UG, UP, AG, and AP) was measured and placed into 250 mL of sterile Thioglycolate enrichment broth as shown in Fig. 1. The broth cultures were incubated either stationary or on an orbital shaker (Crystal Industries, Model SYC-2102A) at 80 rpm at 37 °C for seven days as shown in Fig. 2. After the seven days, the broth cultures were sub-cultured to Trypticase Soy Agar with 5 % sheep blood (BAP) and incubated at 37 °C aerobically and anaerobically. The BAPs were examined at 24 (aerobic) and 48 (anaerobic) hours for microbial growth. This procedure was performed in triplicate for each group.

### Statistical methods

Statistical differences among treatment groups were analyzed by a Welch’s t-test and the Prism 7.02 (GraphPad Prism, San Diego, CA) software (Shreffler & Huecker, 2023). Significance was set at *p* ≤ 0.05. Statistical significance was calculated between UG and UP. The average of the three runs for UG and UP were used. All values were presented as mean ± standard error of the mean (SEM) from three replicate trials.

## Results

### Microbial plate counts

**Fig. 3:**
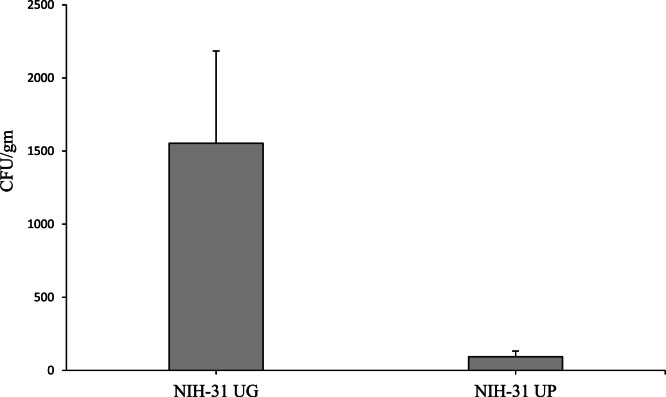
NIH-31 analysis of mean cognitive total plate counts, TPC (CFU/gm) of non-sterile NIH-31 unautoclaved pelleted (UP) and ground (UG) feed incubated aerobically for 7 days at 37 °C. Values are reported as mean ± SEM. Value is not statistically different (*P* > 0.05) when the unsterilized NIH-31 UP and UG are compared with TPC.

The NIH-31 feed was analyzed for total microbial plate counts using the standard microbiological procedures (Carter, 1973; Sanders, 2012) (Table 1). The procedure was repeated in triplicate, and the average of the bacterial plate counts (CFU/gm) was calculated. The UG grew on average 1553 CFU/gm ± SEM aerobically and UP grew on average 93 CFU/gm ± SEM aerobically. While the unautoclaved, ground feed had higher bacterial counts compared to the unautoclaved, pelleted feed, the difference was not significant (*p* = 0.132) in Fig. 3.

**Table 1:** Total plate counts (TPC) are measured in colony forming units (CFUs). The results shown are the averages from the analytical procedure that was ran in triplicate and incubated at 37 °C under aerobic (24 h) environmental conditions.

NIH-31 Feed Groups	TPC 1:10 Dilution		TPC 1:100 Dilution		Environmental Condition
	1 mL x 10	0.1 mL x 100	1 mL x 1000	0.1 mL x 10,000	
UG	TNTC*	1533	5000	0	Aerobic
UP	93	433	1000	0	Aerobic
AG	0	0	0	0	Aerobic
AP	0	0	0	0	Aerobic

⁎ Too numerous to count.

### Sterility testing- stationary versus shaking incubation

The sterility of the autoclaved ground and pelleted NIH-31 diets was tested on the Thioglycolate broth cultures after 7 days of incubation either stationary or on an orbital shaker. The feed pellets in the stationary cultures failed to fully breakdown, whereas the pellets on the shaker were fully broken down. UG and UP were culture positive for bacterial growth on BAP’s aerobically and anaerobically when incubated either stationary or shaking (Table 2). AG and AP were culture negative for bacterial growth on BAPs aerobically and anaerobically.

**Table 2:** Sterility test results using NIH-31 diet to compare shaking and non-shaking test methods. Bacteria growth (positive (+) or negative (-)) are the averages from the analytical procedure ran in triplicate and incubated at 37 °C under aerobic (24 h) and anaerobic (48 h) environmental conditions.

Non shaking technique on blood agar plates (BAPs)		
NIH-31 Feed Groups	Bacteria Growth (BAP)	Environmental Condition
UG	(+)	Aerobic
UG	(+)	Anaerobic
UP	(+)	Aerobic
UP	(+)	Anaerobic
AG	(-)	Aerobic
AG	(-)	Anaerobic
AP	(-)	Aerobic
AP	(-)	Anaerobic

Shaking technique on BAPs		
NIH-31 Feed Groups	Bacteria Growth (BAP)	Environmental Condition

UG	(+)	Aerobic
UG	(+)	Anaerobic
UP	(+)	Aerobic
UP	(+)	Anaerobic
AG	(-)	Aerobic
AG	(-)	Anaerobic
AP	(-)	Aerobic
AP	(-)	Anaerobic

## Discussion

Microbial contamination of both human and animal feed is still a significant health risk, and the surveillance of feed for microbial contamination remains an important step in limiting feed-borne-infections. Although feed manufactures must maintain stringent processes to minimize contamination risk, we should continue microbial contamination testing of non-sterile feeds intended for laboratory animals. Otherwise, we risk animal health and the introduction of an extrinsic variable to research studies that can have effects on the animals’ physiological responses (Kurtz et al., 2020). This variability in-turn can affect research outcomes and study replicability. Recent studies have reported rodent feed as the possible source for Mouse Parvovirus (MPV) (Watson, 2013). Similarly, we recently reported the isolation of *Clostridium perfringens* from several natural ingredient, rodent diets (Johnston et al., 2022). Others have shown that MPV and Mouse Norovirus (MNV) can survive rodent feed pelleting (Adams et al., 2019; Watson, 2013).

The objective of this study was to compare the sensitivity of microbial detection between pelleted and ground rodent feed. This study also investigated whether homogenization of the feed pellets by shaking improves sensitivity for identifying bacteria when conducting microbial sterility testing. Grinding of the unautoclaved NIH-31 diet demonstrated a difference in the microbiological counts between pelleted versus ground feed; however, this difference was not statistically significant due to the small sample size and variability in the replicate counts (Fig. 3). Both the AG and AP were found to be sterile. Our results indicated that grinding pelletized feed, sterilized in a properly functioning autoclave, prior to microbial testing does not improve the microbial testing sensitivity. Thus, reducing the labor, time, and handling contamination risk when performing sterility test. We also demonstrated that mixing the intact feed pellets on an orbital shaker during incubation does not increase the sensitivity of detecting bacteria. The grinding or homogenization of feed prior to microbial testing recommended by the FDA and USDA applies to non-sterile food/feed, and our results do indicate that this process does increase the microbial counts in non-sterile ground NIH-31 feed compared to intact feed pellets. When feed is autoclaved sufficiently, we found that grinding or shaking pelleted rodent feed is not necessary nor does it increase the sensitivity of bacterial detections resulting in reduced testing time, reduced labor, and less risk of sample contamination due to handling.

## Funding

This research was supported by the Intramural Research Program of NIEHS.

## Ethics in publishing statement

This research presents an accurate account of the work performed, all data presented are accurate and methodologies detailed enough to permit others to replicate the work.

This manuscript represents entirely original works and or if work and/or words of others have been used, that this has been appropriately cited or quoted and permission has been obtained where necessary.

This material has not been published in whole or in part elsewhere.

The manuscript is not currently being considered for publication in another journal.

That generative AI and AI-assisted technologies have not been utilized in the writing process or if used, disclosed in the manuscript the use of AI and AI-assisted technologies and a statement will appear in the published work.

That generative AI and AI-assisted technologies have not been used to create or alter images unless specifically used as part of the research design where such use must be described in a reproducible manner in the methods section.

All authors have been personally and actively involved in substantive work leading to the manuscript and will hold themselves jointly and individually responsible for its content.

## Ethics in publishing statement

Our study did not require an ethical board of approval because it did not contain human or animal trials.

## CRediT authorship contribution statement

**Jonathan W. Weeks:** Writing – original draft, Visualization, Validation, Methodology, Investigation, Formal analysis, Conceptualization. **Jacqueline Locklear:** Writing – review & editing, Visualization, Methodology, Conceptualization. **Tanya E. Whiteside:** Writing – review & editing, Visualization, Methodology, Conceptualization. **David M. Kurtz:** Writing – review & editing, Visualization, Methodology, Conceptualization.

## Declaration of competing interest

The authors declare that they have no known competing financial interests or personal relationships that could have appeared to influence the work reported in this paper.
